# Role of host genetics in fibrosis

**DOI:** 10.1186/1755-1536-2-6

**Published:** 2009-12-04

**Authors:** Georgina L Hold, Paraskevi Untiveros, Karin A Saunders, Emad M El-Omar

**Affiliations:** 1 Division of Applied Medicine, Institute of Medical Sciences, University of Aberdeen, Foresterhill, Aberdeen AB25 2ZD, UK

## Abstract

Fibrosis can occur in tissues in response to a variety of stimuli. Following tissue injury, cells undergo transformation or activation from a quiescent to an activated state resulting in tissue remodelling. The fibrogenic process creates a tissue environment that allows inflammatory and matrix-producing cells to invade and proliferate. While this process is important for normal wound healing, chronicity can lead to impaired tissue structure and function.

This review examines the major factors involved in transforming or activating tissues towards fibrosis. The role of genetic variation within individuals affected by fibrosis has not been well described and it is in this context that we have examined the mediators of remodelling, including transforming growth factor-beta, T helper 2 cytokines and matrix metalloproteinases.

Finally we examine the role of Toll-like receptors in fibrosis. The inflammatory phenotype that precedes fibrosis has been associated with Toll-like receptor activation. This is particularly important when considering gastrointestinal and hepatic disease, where inappropriate Toll-like receptor signalling, in response to the local microbe-rich environment, is thought to play an important role.

## Background

Fibrosis is a wound-healing response by which the body attempts to repair itself following injury. Acute and, more commonly, chronic injury from a wide variety of insults leads to organ fibrosis. Organ systems have different cellular and molecular mechanisms that result in fibrosis [1-6]. Fibrous tissue contains extracellular matrix (ECM) but in different ratios to normal tissue. In particular, there is a significantly increased amount of type I collagen, with progressive fibrosis eventually leading to a distortion of the normal organ architecture [7,8]. The distorted architecture, along with loss of normal cellularity, leads to a loss of function of the underlying organ. For example, fibrosis in the liver can interfere with drug metabolism, cause accumulation of toxic metabolites and lead to the synthetic failure of important coagulation factors. In lung tissue, fibrosis leads to poor blood-gas exchange resulting in progressive hypoxia as the disease process advances. There is also strong evidence linking fibrotic progression and angiogenesis [9].

Fibrous tissue is laid down by cells with a mesenchymal-like phenotype. In the liver the hepatic stellate cell (HSC) is the major cell responsible for fibrosis, with activation of HSC being a key fibrotic event, although fibroblasts and bone marrow derived fibrocytes all contribute [10-13]. Fibrosis has been described in virtually every organ and most evidently in the liver. Other organs include the lungs, skin, blood vessels, heart and kidneys. In the UK, excessive alcohol consumption remains the most common cause of hepatic fibrosis. Worldwide chronic hepatitis B and C are the principle factors and are a major cause of morbidity and mortality. At a molecular level, transforming growth factor-beta (TGF-β), tissue inhibitor of metalloproteinases (TIMP) and matrix metalloproteinases (MMP) are the key factors involved in the development of fibrogenesis [14,15]. There are a number of other factors contributing to fibrogenesis that may play more significant roles in particular organs. Reactive oxygen species in lungs and liver [16-18], hypoxia inducible factor in kidneys [4] and angiotensin II in blood vessels [19] are examples. In this review we focus on TGF-β, IL-13, TIMP and MMP, the major factors implicated in fibrogenesis.

## The role of TGF-β in fibrogenesis

The elucidation of the pathways involved in TGF-β signal transduction has provided new therapeutic targets for the prevention or treatment of fibrosis. TGB-β is a pleiotrophic growth factor which is involved in fibroblast chemotaxis and proliferation. Transient TGB-β1 activity is known to participate in the repair and regeneration of tissues. However, persistent TGB-β1 function induces excessive fibrosis and, ultimately, scarring of both skin and internal organs [20]. TGB-β promotes production of several ECM proteins, including type I collagen, by stimulating its gene transcription. It also influences MMP/TIMP expression and T cell function and, thus, inflammatory reactions are also influenced by TGF-β [21]. *COL1A1 *and *COL1A2 *are the genes encoding the polypeptides which form Type I collagen which is the most abundant product of fibrosis, with the development of fibrosis corresponding with an increased rate of the transcription of these two genes [22-24]. Interestingly, enhancer sequences for *COL1A1 *include binding sites for Smad, Sp1, p38 MAPK and NF-1 [25]. The enhancer region for *COL1A2 *contains corresponding regions for Smad, Sp1, AP-1 [26-29]. These regulatory gene proteins are known to enhance the effects of TGF-β *COL1A2 *expression along with the cAMP response element binding protein (CBP) and p300 coactivators [30].

**Figure 1:**
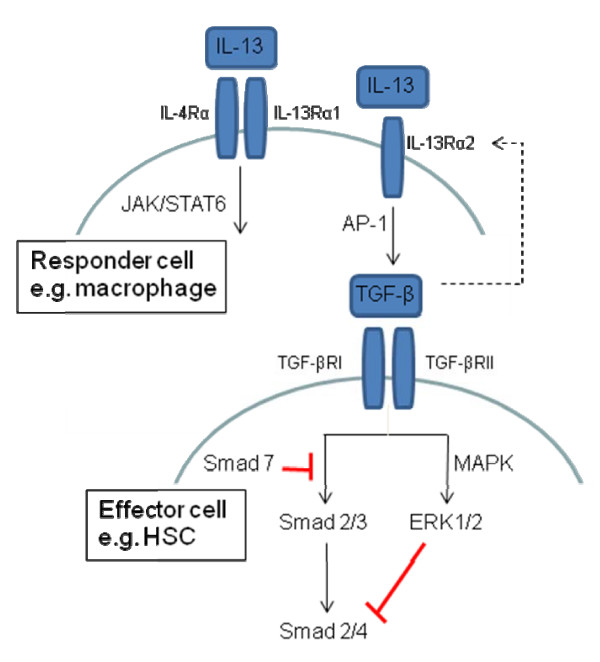
**Interleukin (IL)-13/transforming growth factor (TGF)-β signalling pathways**. IL-13 signalling via IL-4Rα/IL-13Rα1 occurs via the JAK/STAT6 pathway and can cause increased collagen production from fibroblasts, recruit immune effector cells and enhance chemokine expression. Signalling via IL-13Rα2 in macrophages activates AP-1 inducing TGF-β secretion. Similarly IL-13Rα2 expression can be enhanced by TGF-β in hepatic stellate cells. TGF- β activates two serine/threonine kinase receptors and signals through Smad phosphorylation. TGF-β can also activate mitogen activated protein kinase signalling.

TGF-β is secreted in inactive form which is then activated following proteolysis [21]. Once active, TGF-β is free to bind to its receptors and the resulting signal transduction pathway in the cytoplasm involves activation/translocation of Smad (a family of gene regulatory proteins) to the nucleus (Figure 1). Smad 1, 2, 3, 5 and 8, also known as receptor associated Smads (R-Smads), become phosphorylated when the TGF-β and BMP receptors are activated. Once phosphorylated, these R-Smads dissociate from the receptor and must complex with Smad 4 before they can translocate to the nucleus. Transcription of target genes is then achieved when the phosphorylated Smad complex binds to a specific area of DNA. A number of co-activators (for example, CBP and p300) and transcription factors are also involved in modulating sites of transcription [21]. Smad 6 and 7, unlike other members of the Smad family, prevent phosphorylation and thus activation of these Smad complexes are involved in TGF-β receptor degradation [31].

In addition to activation of the Smad signal transduction pathway, TGF-β activates the mitogen activated protein kinase (MAPK) family (Figure 1) [21]. The TGF-β response is enhanced or inhibited depending on the particular MAPK pathway involved. P38 MAPK and c-Jun N-terminal kinase (JNK) can activate Smad 3 [32,33] and p38 MAPK also strengthens interaction between Smad3 and coactivators [34]. Decreased Type I collagen expression has generally been demonstrated with p38 MAPK inhibition [32,33,35]. The final MAPK pathways - extracellular signal regulated kinase (ERK) - inhibit Smad signal transduction, as well as BMP, and Smad1 effects on transcription [36]. The impact of ERK on collagen gene transcription is cell specific with ERK causing increased collagen production in some cells and decreased production in others [37].

## IL-13 and fibrosis

The cytokine environment also plays a role in tissue remodelling and it seems likely that they can influence the phenotypic changes seen in different cell types in fibrotic tissue. IL-13 is a Th2 cytokine that is known to induce fibrosis through the regulation of TGF-β1 production and activation [38,39]. IL-13 binds to two primary receptor chains IL-13Ralpha1 and IL-13Ralpha2 (Figure 1). IL-13Ralpha1 is expressed in healthy tissue and binds IL-13 through the formation of a heterodimer complex with IL-4Ralpha chain. This complex formation culminates in signal transduction via the JAK/STAT6 pathway [40,41]. IL-13Ralpha2, on the other hand, is only marginally expressed in normal healthy tissue and over-expressed in several abnormal cells including cancerous and fibrotic. However, it can bind IL-13 with a high affinity with mediation of signal transduction, thought to be STAT6 independent, signalling instead through the AP-1 pathway (Figure 1) [39]. Due to its lack of expression in normal tissue and over expression in cancer cells and during fibrosis, it has been suggested that IL-13Ralpha2 chain may serve as a novel biomarker for diseased cells and a target for receptor-directed therapeutics [39,42,43].

Fibroblast IL-13Ralpha2 expression has been reported in several fibrotic diseases including idiopathic interstitial pneumonia, schistosomiasis and non-alcoholic steatohepatitis [44-46]. Within the context of hepatic disease, IL-13Ralpha2 is expressed in activated HSCs but not quiescent HSCs, with expression strongly induced by both TGF-β1 and also TNF-α [46]. Interestingly, TGF-β independent IL-13 induced fibrosis has also been identified, within the context of parasitic disease, with IL-13Ralpha2 over expression acting as a soluble decoy receptor and ultimately decreasing fibrosis [47].

## MMPs and TIMPs

MMPs and TIMPs are also responsible for maintaining integrity of the ECM. There are many different types of metalloproteinases, some of which are specific and others that are less discriminating to ECM substrates. Their presence is required to degrade the wide variety of components of the ECM. Excessive catabolism of ECM is kept in check with several mechanisms, including the secretion of TIMPs. TIMPs work by binding to MMPs thereby blocking their activity.

Expression of MMP subtype is tissue dependent with differences in amino acid sequence of MMPs seen amongst animal species [48]. MMP-1 is significantly expressed by several types of cells including the HSC. MMP-13 is the rodent homologue of human MMP-1 [49-51]. Following liver injury, elevated levels of MMP-1 and MMP-13 RNA have found in human and rodent liver tissue respectively [51-54]. Elevated metalloproteinase expression, in acute liver injury has been shown to occur for only a short period post insult [54] and, in chronic liver injury, is limited to the period of fibrogenesis [55]. After liver injury, HSCs become activated and express MMP-2 and MMP-14 [50,56]. Elevated MMP-2 and MMP-14 levels have been demonstrated in fibrotic and cirrhotic liver tissue, with the exception of HCV induced cirrhosis [57,58]. MMP-2 promotes proliferation and migration of the hepatic stellate cells and its activation is dependent on MMP-14 [59]. Both TIMP-1 and TIMP-2 can inhibit MMP 2.

There is brief expression of MMP-3 following HSC activation and acute toxic liver injury. The most important role MMP-3 is known to play in fibrosis is through cleavage of MMP precursors such as MMP-1, -3, -7, -8, -9 and -13 to their active forms [60-65]. However, there is contradictory evidence in the literature on whether MMP-3 expression is increased or decreased in chronic liver injury [66-68]. Similarly, variable results have been obtained for MMP-9 expression in both acute and chronic liver injury [57,69-71]. IL-13 is a potent inducer of MMP-9. The role of MMP-9 in fibrogenesis is thought to primarily involve activation of TGF-β [72]. This is significant as initial collagen production by the HSC is stimulated by TGF-β [73].

TIMP-1 and TIMP-2 are the main TIMPs associated with fibrosis. In the liver, these TIMPs are primarily produced by HSCs, although other cells also contribute to TIMP production [74]. In hepatitis C infection, the degree of hepatic fibrosis is correlated with the level of TIMP-1 mRNA and protein [53]. In addition to binding, and thereby inhibiting matrix metalloproteinases, TIMP-1 also prevents apoptosis of HSCs [75,76]. Elevated levels of TIMP-2 in serum and liver mRNA are found in human hepatitis C virus (HCV) liver disease. However, fibrosis does not have to be present for raised TIMP-2 levels to be detectable in HCV patients [77,78]. In rodents, TIMP-2 mRNA reaches its highest levels in acute toxicity within 3 days, whereas it is not increased with chronic toxic liver injury [57,70,79].

## Host genetic factors and fibrosis

Before discussing the role of host genetic factors in fibrosis, it is essential to establish some basic principles of genetic epidemiology and the limitations of studying genetic polymorphisms in the context of complex multifactorial human diseases [80]. The overwhelming majority of polymorphisms studied are single nucleotide polymorphisms (SNPs) that occur with a frequency of >1% in the normal population (in contrast to 'mutations' that occur with a frequency of <1%). It is estimated that the human genome contains up to 10 million SNPs, although not all have thus far been identified. Most SNPs are located within non-coding regions of the genome. However, of those that are located within coding sequence, most are non-synonymous and are not associated with the alteration of the amino acid sequence rendering them of no functional consequence. Other types of genetic variation include deletion and insertion polymorphisms and microsatellite repeat polymorphisms.

There has been an exponential rise in the number of published genetic association studies. Quite often, a report of a single genetic marker is published with great promise, only to be followed by several negative studies that fail to reproduce the original observation. There is no doubt that the strategy of genetic association studies could be a powerful tool for dissecting human diseases, provided certain principles are observed in order to minimize the chances of false positive, and negative, reports. The most important of these principles include: rigorous definition of disease phenotype; choice of candidate genes that are plausibly linked to the pathophysiology of the disease under study; selection of polymorphisms with known (or at least potentially) functional consequences; choice of genetic markers that are reasonably frequent in the population under study (variant allele frequency of at least 5%); appropriate selection of controls that are matched for ethnicity, age, gender and environmental exposures; and design of studies that are adequately powered to produce a valid result. Even then, the statistical analyses of such studies have to take into account the real problem of false positive results by using multiple testing. Appropriate corrections for multiple testing have to be applied or, alternatively, the positive findings should be regarded as preliminary and should be validated in an independent set of cases and controls. Finally, the genetic epidemiology has to involve basic science in order to unravel and validate the molecular mechanisms involved. Adherence to these basic principles will ensure that false positive trails are minimized and will offer a true opportunity to understand the complex multifactorial human diseases. There has never been a better time to stress the necessity for an adherence to these principles, as the advancement in genotyping technology has made possible the annotation of the entire human genome. Current technology allows us to genotype up to 500,000 SNPs in one run. We have witnessed a shift towards these so-called whole genome association studies, where large multi-centre consortia attempt to examine a very large number of cases and controls for a particular disease. The power of these studies allows for an exploratory phase where thousands of SNPs are examined and a validation phase that attempts to replicate positive associations independently.

Having set the background to the study of genetic polymorphisms, we can now examine the role of these in the context of fibrosis. Pathogenic fibrosis typically results from chronic inflammatory reactions, many of which will be triggered by an infectious agent or a chemical assault which drives the chronic inflammation and the subsequent development of fibrosis. The role of polymorphisms in several cytokine genes has been examined in the context of fibrotic disease, often with conflicting results. We will concentrate on genetic markers that have relevance to pathogenesis of fibrotic diseases and will only consider markers that satisfy the criteria listed above.

Perhaps the most relevant gene in the context of fibrosis is TGF-β. Several SNPs have been identified in this gene and some are associated with elevated TGF-β1 concentrations in human plasma [81-83]. However, only SNPs within the coding region of TGF-β1 (Leu10Pro and Arg25Pro) have been shown to be associated with increased fibrotic risk [84-88] (Table 1). Gewaltig *et al. *reported that the carriage of at least one Pro at codons 10 and/or 25 was significantly associated with a faster progression of hepatic fibrosis following chronic hepatitis C infection. The fibrosis progression rate of patients with genotypes ^10^LeuPro and ^10^ProPro was almost three times as fast as those having genotype ^10^LeuLeu. Stage and histological activity grade of fibrosis in ^25^ArgPro in comparison to ^25^ArgArg were also higher [84]. Tag *et al. *were able to reproduce similar findings reporting an increased risk of higher grades of fibrosis in carriers of the ^25^ArgPro genotype [85]. However, these were small studies and findings from other groups have either failed to replicate the associations or reported opposite associations. For example, Powell *et al. *showed that the ^25^ArgArg genotype was associated with increased risk of hepatic fibrosis following HCV infection [87]. The same polymorphisms have been addressed in other hepatic disorders. Österreicher *et al. *studied the role of host genetic factors in the progression of hereditary haemochromatosis and showed that the ^25^ArgPro genotype increased the risk of cirrhosis by nearly threefold compared to ^25^ArgArg genotype [86]. The direction of association is similar to that reported by Gewaltig and Tag *et al. *but the studies remain small and require definitive validation in larger case control studies.

**Table 1:** Genetic polymorphisms with relevance to fibrosis risk.

Gene	Known variation	Effect	Reference
TGF beta	Leu10Pro	LeuLeu showed a slow progression of fibrosis Carriage of Pro associated with faster fibrotic progression	[84-88]
		
	Arg25Pro	Carriage of Pro associated with faster fibrotic progression	
Angiotensin	G-6A	Carriage of AA genotype associated with increased risk of fibrosis	[87]

TNF-alpha	G-308A	A allele associated with hepatic fibrosis, hepatic cancer, fibrosing alveolitis	[91,93,136,137]

IL-10	C-592A/-819/G-1082A	Carriage of ATA associated with ALD and HCV induced fibrosis	[99,100,138]

IL-1	IL-1RNC +2018T	T allele associated with increased risk of fibrosing alveolitis	[91]

IFN-gamma	T+874A	T allele associated with higher rate of liver cirrhosis following Hep C infection	[103,104]

CC chemokine receptor 5 (CCR5)	insertion/deletion (Δ32)	Carriage of Δ32 associated with severe fibrosis	[107]

MCP-2	Q46K	Carriage of the K variant is associated with more severe fibrosis	[107]

MCP-1	G-2518A	Carriage of G allele associated with increased risk of hepatic inflammation and fibrosis	[109]

Haemochromatosis gene (*HFE*)	G+845A C+187G	Heterozygous genotypes associated with increased inflammation and fibrosis	[110,111]

Myeloperoxidase (MPO)	G-463A	Minor allele associated with increased risk of advanced fibrosis in CHC patients	[112]

low density lipoprotein receptor (LDLR)	G+1170A	Carriage of G associated with increased risk of fibrosis	[113]

Apolipoprotein E (Apo E)	E4 allele	Carriage of E4 allele associated with protection against HCV induced liver damage	[114]

DDX5 DEAD box polypeptide 5 and POLG2 SNPs		Minor allele associated with increased risk of advanced fibrosis in CHC patients	[139]

CD14	C-159T	T allele associated with higher levels of acute phase proteins and advanced ALD	[140]

TLR4	D299G T399I	Both variant allele confer protection against fibrosis	[141,142]

CHC, chronic hepatitis cancer; ALC, alcoholic liver cirrhosis; ALD, alcoholic liver disease; HCV, hepatitis C virus

TGF-β1 production is also known to be enhanced by angiotensin II, the principal effector molecule of the renin-angiotensin system. A statistically significant relationship was also seen between the polymorphism in the promoter region of the angiotensinogen gene (*AT-6*) and the stage of hepatic fibrosis [87]. Individuals with the adenine/adenine homozygous genotype were more likely to have increased hepatic fibrosis compared with individuals inheriting the adenine/guanine or the guanine/guanine homozygous genotype (Table 1).

The *TNF-A*-308 G > A polymorphism is known to be involved in a number of inflammatory conditions. Carriage of the pro-inflammatory A allele has been shown to increase the odds ratio for severe disease in both hepatic fibrosis and also fibrosing alveolitis (Table 1). Yee *et al. *reported carriage of the -308A allele was associated with a fivefold increased risk of cirrhosis following HCV infection [89]. These findings were reported by Kusumoto *et al.*, with carriage of 'A' at TNF-α -238 or -308 correlating with significantly higher serum levels of Type IV collagen 7S, which is a marker for advanced hepatic fibrosis [90]. However, other reports failed to confirm these associations. Carriage of TNF-*A*-308 A has also been assessed within the context of fibrosing alveolitis in several small studies. Whyte *et al. *assessed the frequency of the polymorphism in two independent case-control studies, one English and one Italian, and showed a significant association of TNF-*A*-308 A carriage with increased risk of fibrosing alveolitis in the Italian, but not the English, study [91]. Studies by Pantelidis *et al. *and Riha *et al. *confirmed this association but the findings require confirmation in a much larger study with appropriately matched controls [92,93].

Other cytokine genes in which genetic variation has been examined within the context of fibrotic disease include interleukin-10, interferon-gamma and the interleukin (IL)-1 receptor antagonist. IL-10 is an anti-inflammatory Th2 cytokine that down regulates IL-1β, TNF-α, interferon-γ and other pro-inflammatory cytokines and has a modulatory effect on hepatic fibrogenesis. IL-10 levels differ widely between individuals, possibly because of polymorphisms in the promoter region of the *IL-10 *gene at positions -592, -819 and -1082 [94,95]. Promoter polymorphisms have been associated with several inflammatory conditions including hepatitis B virus-induced hepatocellular carcinoma and other cancers [96,97]. We have previously reported that homozygosity for the low-IL-10 *ATA *haplotype increased the risk of non-cardia gastric cancer [98]. *IL-10 *SNPs have been studied in the context of hepatic fibrosis. An early study by Powell *et al. *did not show a correlation between stages of HCV induced fibrosis and *IL-10 *promoter polymorphisms [87]. However, a study published the same year, looking at their role in alcoholic liver disease induced fibrosis, indicated a strong association between possession of the A allele at position -592 in the *IL-10 *promoter region and fibrosis [99]. It was subsequently suggested that defining disease progression would possibly be more appropriate based on the speed of fibrotic development (that is, fast versus slow). A subsequent study by Knapp *et al. *looking at HCV-induced fibrosis, showed a higher frequency of the low IL-10 producing haplotypes (ACC/ACC and ATA/ATA) in patients termed 'fast fibrosers' [100] (Table 1).

Polymorphisms in interleukin-1 that have been assessed in the context of fibrotic disease are mainly related to the IL-1 receptor antagonist. Whyte and colleagues looked at the *IL-1RN *polymorphism at C+2018T in two European cohort studies of pulmonary fibrosis along with the previously mentioned *TNF-A*-308 G > A polymorphism [91]. As with the *TNF-A*-308 G > A polymorphism, carriage of the rarer T allele at *IL-1RN *+2018 was associated with an increased risk of fibrosing alveolitis in the Italian but not the English cohort. This is again possibly due to small study numbers. A variable tandem repeat genetic variant in intron 2 of *IL-1RN *which is in strong linkage disequilibrium with the C+2018T has also been studied. However, no association has been defined in either hepatic or pulmonary fibrosis [93,101,102]. Carriers of interfernon (*IFN)-G *+874 T allele have also been shown to have a significantly higher rate of liver cirrhosis and early recurrent hepatitis C after transplantation [103,104]. The AA genotype is associated with the low levels of IFN-gamma production which is thought to inhibit the appropriate level of T-helper (Th1) response needed to combat HCV viral load and subsequent disease progression [105,106].

Assessment of genetic variation in chemokine receptors has also been studied in the context of HCV-induced hepatic fibrosis. Hellier *et al. *reported a significant association between severe HCV-induced hepatic fibrosis and carriage of both CCR5 Δ32 and the K variant of the MCP-2 Q46K polymorphism [107]. In total 20 polymorphisms in seven CC chemokines and their receptors were assessed in the study which comprised 672 patients. Both chemokines are involved in T cell recruitment/migration and processes relevant to HCV clearance or persistence. Particularly in relation to CCR5 Δ32, for which functional data is available, carriage of the 32 bp deletion results in a non-functional protein which will impact on viral persistence [108]. The -2518 MCP-1 promoter polymorphism has also been shown to be a risk factor for HCV induced hepatic fibrosis. An elegant study by Muhlbauer *et al. *showed that carriage of MCP-1 -2518 G allele, which is associated with increased MCP-1 levels, was associated with more advanced fibrosis and severe inflammation [109]. The study also demonstrated, for the first time, an association of the MCP-1 polymorphism with MCP-1 tissue levels.

Polymorphisms involved in other cellular processes important in hepatic fibrotic development have also been studied. Mutations within the haemochromatosis (*HFE*) gene involved in iron storage and accumulation have been shown to be associated with higher grades of inflammation and more severe hepatic fibrosis, although these findings were not replicated in other published studies [110,111]. Potential explanations for the lack of validation include: small sample size; different histological scores for hepatic fibrosis; ethnicity; population stratification; and uncontrolled variables associated with disease progression. A promoter polymorphism within the myeloperoxidase gene which is involved in activation of HSCs and the production of ECM-*MPO *G-463A has also been shown to be associated with advanced fibrosis when the variant A allele was present [112]. Polymorphisms in genes involved in lipid transportation have also been assessed within the context of HCV-induced liver fibrosis. These pathways are thought to promote viral endocytosis. Carriage of the G allele of the low density lipoprotein receptor polymorphism - G+1170A - has been shown to render patients more susceptible to developing severe HCV-induced fibrosis [113]. Conversely, carriage of the apolipoprotein (apoE) E4 allele has been shown to protect against severe liver damage induced by HCV [114] (Table 1).

## Toll-like receptors (TLRs) and fibrosis

There is growing interest in the role of the innate immune system, especially TLRs, as regulators of wound healing and especially fibrosis. TLRs are a highly conserved family of germline-encoded receptors that recognize structural motifs expressed by bacteria, viruses and fungi. Stimulation of TLRs by these ligands activates numerous signalling cascades which ultimately culminate in proinflammatory cytokine production and other immune responses, including cell survival and apoptosis [115]. Currently 10 human TLRs have been identified, each with different ligand specificity. TLR4 is known as the lipopolysaccharide (LPS) receptor, due to the original reports which demonstrated the relationship between TLR4 and LPS recognition [116,117]. LPS - or endotoxin - a component of the Gram-negative bacterium outer membrane is are now known to be one of a collection of ligands that is recognized by TLR4. However, it is known that TLR4 (and possibly other TLRs) can detect other exogenous as well as endogenous ligands, many of which are most abundant during tissue injury such as hyaluronan, fibronectin S100 proteins and heat shock proteins 60 and 70 [118]. Along with TLR4, TLRs 1, 2, 5, 6 and 9 are involved in bacterial recognition. TLR 1, 2 and 6 recognize lipoprotein from Gram-positive bacteria and TLR5 is involved in bacterial flagellin sensing. TLR9 recognizes non-methylated CpG-containing DNA from bacteria. In contrast, TLR 3, 7, 8 and 9 recognize viral nucleic acids. TLRs although similar in their structure with a leucine-rich repeat domain and a Toll/IL-1 receptor (TIR) domain are separated on the basis of their cellular location with TLR 1, 2, 4, 5 and 6 located on the cell surface, whilst the others are associated with endosomal/lysosomal compartments where the possibility of encountering host DNA and therefore eliciting self-recognition is reduced [119].

**Figure 2:**
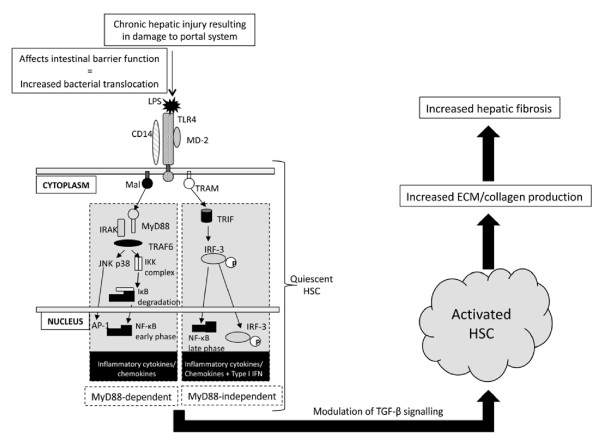
**Signalling pathways triggered by TLR4 activation as a result of damage to the portal system**. Altered barrier function resulting in increased bacterial translocation allows bacterial products including lipopolysaccharide (LPS) to activate hepatic stellate cells (HSCs) through Toll-like receptors (TLRs) (TLR4 shown as an example TLR). Activation of TLR4 through LPS binding initiates numerous signalling cascades which culminate in activation of transcription factors NF-κB and AP-1 and production of inflammatory cytokines/chemokines and immune mediators. This activation sensitises HSCs to the effects of transforming growth factor TGF-β which ultimately results in HSC activation and increased extracellular matrix/collagen production resulting in increased hepatic fibrosis.

Following ligand binding, TLR signalling cascades are initiated from the TIR domain and many of the signalling molecules that mediate the intracellular response are common between the TLRs [120]. TLR signalling has been divided into MyD88-dependent and MyD88-independent (TRIF dependent) pathways (Figure 2). MyD88-dependent signalling culminates in activation of the transcription factors NF-kB and AP-1 (via downstream MAPK pathways) and the production of numerous pro-inflammatory cytokines and immune mediators. These transcription factors are also activated via MyD88-independent signalling but their activation is slightly delayed [121]. All TLRs with the exception of TLR3 signal via the MyD88-dependent signalling pathway. MyD88-independent signalling is involved in the induction of interferon-inducible genes including IRF3 which are important for anti-viral and anti-bacterial responses [122,123]. TLR4 is the only TLR known to utilize both the MyD88-dependent and independent pathways [124,125].

Although all immune cells express TLRs, these receptors are also present on other classes of cell. Nevertheless, the ability of different cell types to recognize and respond to microbial ligands differs. Generally, TLR expression on immune cells is there as the archetypical response to infection. TLR activation on other cell types, including epithelial cells, whilst contributing to the immune response has also been suggested to lead to tissue scarring and fibrosis [126,127].

Impaired TLR4 and nine responses through defective signalling, and also the presence of genetic variations, have been shown to reduce hepatic fibrosis [128-130]. A number of *in vivo *observations also support a role for TLRs in promoting fibrogenesis, although this has only been studied within the context of hepatic disease. It has also been shown that the intestinal microbiota is at least in part responsible for activating TLR4 containing cells within the liver, especially quiescent HSCs ultimately evoking hepatic fibrogenesis through modulation of TGF-β signalling [128,131-133]. TLR induced activation of p38 MAPK and JNK has also been shown to be involved in increased production of collagen by HSCs [134]. The link between the intestinal microbiota and hepatic TLR activation is through the portal vein. The most likely explanation is that damage incurred to the portal system, during chronic hepatic injury, affects the intestinal barrier function allowing increased bacterial translocation [128,129] (Figure 2). This view is supported by studies using gut-sterilized mice that have shown a strong reduction in fibrogenesis compared to conventional mice [135].

## Conclusion

Fibrosis can occur in almost any tissue type, with analysis of the cellular and molecular mechanisms showing similarities irrespective of location. Trauma/insult usually through exogenous stimuli, chemical or microbiological, results in innate immune cell activation which triggers a chronic inflammatory response that is central to fibrotic perpetuation. TGF-β plays a pivotal role in the fibrotic development through its influence on MMP/TIMP expression, T cell function and also ECM production. However, further studies are required in order to fully understand the complex relationship. The situation is further complicated by the contribution of host genetic polymorphisms to an individual's risk.

Chronic inflammation, whether caused by microbes, chemical or physical trauma favours fibrotic progression. A series of intricate host responses are initiated that, on one hand, are attempting to initiate repair of the tissue damage through resolution of the inflammation, whilst also trying to eliminate the infection. The Th2-type cytokine response (typified by IL-13) seen in fibrosis is pivotal to this as is the increasing understanding of TLR signalling and the impact of genetic polymorphisms on these systems. Therapies aimed at suppressing TLR4 signalling, either through preventing LPS release or TLR4 inhibition, is already being considered in the context of hepatic fibrosis, although other fibrotic targets are also under investigation. Understanding the interplay between trauma stimuli and tissue repair is fundamental to resolving the complex interplay between the causes of chronic inflammation and the host's genetic disposition to fibrotic progression, which will aid the development of new and more effective anti-fibrotic strategies in the future.

## Abbreviations

CBP: cAMP response element binding protein; ECM: extracellular matrix; ERK: extracellular signal related kinase; HCV: hepatitis C virus; HFE: haemochromatosis; HSC: hepatic stellate cell; IFN: interferon; IL: interleukin; JNK: c-Jun N-terminal kinase; LPS: lipopolysaccharide; MAPK: mitogen activated protein kinase; MMP: matrix metalloproteinase; R-Smad: receptor associated Smad; SNP: single nucleotide polymorphism; TGF: transforming growth factor; TIMP: tissue inhibitor of metalloproteinase; TIR: Toll/IL-1 receptor; TLR: Toll-like receptor.

## Competing interests

The authors declare that they have no competing interests.
